# American Proctologist Society

**Published:** 1917-12

**Authors:** 


### AMERICAN PROTOLOGIC SOCIETY.

**Nineteenth Annual Meeting, Held at New York, N.Y., June, 4 and 5, 1917.**

**OFFICERS ELECTED FOR THE ENSUING YEAR:**

President, Jerome M. Lynch, M.D., New York, N. Y.

Vice-President, E. H. Terrell, M.D., Richmond, Va.

Secretary-Treasurer, Collier F. Martin, M.D., Philadelphia,

**Executive Council**:—Alfred J. Zobel, M.D., San Francisco, Cal.; Jerome M. Lynch, M.D., New York, N. Y.; T. Chittenden Hall, M.D., Boston, Mass.; Collier F. Martin, M.D., Philadelphia, Pa.

